# Interference Cancellation Based Spectrum Sharing for Massive MIMO Communication Systems

**DOI:** 10.3390/s21113584

**Published:** 2021-05-21

**Authors:** Milembolo Miantezila Junior, Bin Guo, Chenjie Zhang, Xuemei Bai

**Affiliations:** School of Electronics and Information Engineering, Changchun University of Science and Technology, Changchun 130022, China; 2018300111@mails.cust.edu.cn (M.M.J.); zhangcj@cust.edu.cn (C.Z.); baixm@cust.edu.cn (X.B.)

**Keywords:** interference cancellation, spectrum sharing, massive MIMO, signal detection, channel optimization

## Abstract

Cellular network operators are predicting an increase in space of more than 200 percent to carry the move and tremendous increase of total users in data traffic. The growing of investments in infrastructure such as a large number of small cells, particularly the technologies such as LTE-Advanced and 6G Technology, can assist in mitigating this challenge moderately. In this paper, we suggest a projection study in spectrum sharing of radar multi-input and multi-output, and mobile LTE multi-input multi-output communication systems near *m* base stations (BS). The radar multi-input multi-output and mobile LTE communication systems split different interference channels. The new approach based on radar projection signal detection has been proposed for free interference disturbance channel with radar multi-input multi-output and mobile LTE multi-input multi-output by using a new proposed interference cancellation algorithm. We chose the channel of interference with the best free channel, and the detected signal of radar was projected to null space. The goal is to remove all interferences from the radar multi-input multi-output and to cancel any disturbance sources from a chosen mobile Communication Base Station. The experimental results showed that the new approach performs very well and can optimize Spectrum Access.

## 1. Introduction

In recent years, Radar and Mobile communication systems have been exposed to situations in which the two systems have to share the same spectrum. This has opened the flow gate to research interest and scientific dissertation. Over a decade, spectra were mainly shared within the system by applying opportunistic techniques through cognitive radio [1,2]. These techniques were made feasible through spectrum sensing [3] and data management localization [4] by combining the two techniques through radio mapping [5]. Recently, there is improvement in co-existence spectrum sharing with a secondary network element [6,7]. However, in co-channel interference (CCI), spectrum sharing for mobile communication systems and the radars have received very little attention. This is often due to policy problems and spectrum law enforcement challenges. A decade ago, commercial mobile services were not permitted to share spectrum bands with radar communications systems. This is mainly as a result of the interference disturbance that the radar may cause to the mobile system [8]. The United States, through the Federal Communications Commission, recently suggested the use of the 3550–3650 MHz for business broadband [9]. This band, according to these authors, must be shared by satellite, radar and commercial communications systems [8,10]. The radio frequency (RF) spectrum will be shared among many different systems, including radar and cellular systems, because in the future it will be very important to access the interference scenario of these communications systems. Certainly, radars will cause interference to communications systems and vice versa if proper interference mitigation methods and novel spectrum sharing algorithm are not deployed. Meanwhile, considerable improvement has been noted recently in literature regarding interference-mitigation-based spectrum sharing between radar and commercial communications systems [11]. An interference-mitigation technique for radar and commercial communications systems based on a primal-dual subgradient ascent method has been proposed. This tends to find the maximum data transmission strategy for communications and the optimal information extraction waveform for radar. These systems perform well by minimizing target parameter estimation error of radar and data estimation error of communications. But it is challenged by the limitation of the Lagrange function of unique optimal weight, convexity, and its complexity on evaluating the duality [12]. In addition, by considering the sensitivity of the radar system, the wireless communication signal may deteriorate the radar detection performance, as emphasized by [13]. In works conducted by [14], it was revealed that an average intersystem interfence at the radar system that goes from commercial base stations can be presented mathematically with a zone of exclusion. The commercial base stations’ communications systems are assumed to be equipped with many antennas, and the locations of cellular base stations are analyzed as a Poisson point process. A technical method based on random chance-constrained optimization has been presented in a study conducted by [15] to ensure a minimum performance of radar systems, while maximizing the performance of cellular systems. Particularly, the transmit power adaptation of the commercial cellular base stations were considered. In a study by [16], radar and communication systems are both optimized to maximize the probability of detection of radar while guaranteeing the transmission power budget of the base station and signal- to-interference plus noise ratio of communications systems. Moreover, in the same study, a method based more on the view of beamforming optimization constructive interference was proposed [16]. Only the base station’s beamforming is optimized to minimize transmission power while ensuring the received noise ratio at user equipment (UE) and interference threshold to radar or to minimize interference to radar subject to receive noise ratio constraint. However, in these two studies [16,17] only a downlink communication scenario is considered. However, there is a high probability that radar can be affected by uplink communication signals when the radar is deployed near the user equipment. An interference-cancellation-based interference alignment technique in wireless communication systems was proposed by [18,19]. The main idea of interference alignment is to confine interference from other user equipment into a predefined linear space at the receiver on the UE of interest and to separate the desired signal space from the interference space. Additionally, an opportunistic interference-alignment method was proposed for effectively combining the interference alignment (OIA) technique with a UE scheduling approach for both multiuser downlink and uplink networks communications [20,21,22,23,24,25,26,27], and all this work is cellular-communication-centric. An opportunistic interference alignment approach for spectrum-shared radar and uplink cellular communications systems was proposed in [28], where both systems were equipped with multiple antennas. To achieve good performance, the author put more focus on the uplink of the user equipment (UE) of the base stations, rather than the radar that carries heavy frequencies and often causes disturbance in the sharing performance [8]. 

In this paper, we addressed spatial technique on spectrum access for massive multi-input multi-output radar and massive multi-input multi-output mobile communications systems by considering numerous numbers of the base stations. Here the radar and mobile communication systems exchange much interferences on each other. We then proceeded on the estimation of radar-detected signals according to the null space projection channel on radar and mobile systems by applying an interference-channel-collection estimation. Our objective is to analyze the cancellation of all interference schemes on the space. The selection of the best cancellation channel is made possible based on the maximum estimation of mathematical null space and radar projection wave into the cancellation channel. In the present study, we focused more on the radar. For instance, often in the scenario of radar and mobile communications systems, it is obvious that the radar causes more harmful interferences to the long-term evolution (LTE) communications systems, and this is often due to its high frequency range. At the same time, delicate analyses have to be performed so that losses on the radar side can be reduced significantly by thoroughly choosing the best channel and preserving the $i^{th}$ mobile communication base station. Through a systematical inquiry and analytical results, we demonstrate that the dropping of the radar scheme is smaller when it comes to choosing the appropriate channel of interference and handed down to choose the good channel that the radar signals’ detection is estimated. Secondly, we have discussed the challenges of localizing a targeted point of a radar that has spread its waves in the null space interference channel. Obviously, our objective here is to reduce interference on the LTE cellular network. Our scenario is a radar massive multiple-input multiple-output and an LTE mobile massive multiple-input multiple-output. We consider that the wireless mobile cellular system has many base stations. Two spectrum access methods were selected for this purpose. First, we consider the case where on the radar side the accessible degrees of freedom are not sufficient to localize the chosen targeted point and reduce the interferences of the mobile base stations at the same time. Here, we chose one cellular mobile base station as a sample, to do our projection optimization, with a consideration of maintaining as little degradation as possible. Secondly, we considered a scenario where on the radar side, the accessible degrees of freedom are sufficient to localize the chosen targeted point and reduce the interferences of the mobile base stations at the same time. We analyzed the proficiency of the chosen target detection projection wave and performed hand-to-hand comparisons of the two waves. We get the advantage of the use of the generalized likelihood ratio (GLR) for its flexibility and less computation to perform the detection and to obtain test statistics of the null space projection and orthogonal wave signal. The signal target detector execution for both waves was investigated based on theoretical level as well as practical level throughout Monte Carlo simulations.

The present work is organized as follows. In Section 2 we talk about massive multi-input multi-output (MIMO) radar, selection of channel target, orthogonal waves signal, channel interference, the chosen mobile system model, the cooperative RF environment, and architecture. We have analyzed spectrum sharing for both massive MIMO radar and mobile communications systems. Its performance includes matrix projection and a target Detection Decision Test.

In Section 3, we talk about numerical results and analyses. In Section 4, we talk about projection algorithm and discussion. Section 5 is the conclusion of the paper.

## 2. Materials and Methods

### 2.1. System Model

In this section, we presented the chosen detection point target of MIMO radar, in a far-end site, the orthogonal waves, interference channel, and massive mobile cellular network. In the present work, we have considered a radar that is a colocation massive multi-input multi-output radar with M variable of transmitter and receiver antennas grouped inside a military base station. We consider that our colocated multi-input multi-output radar antenna array is half observation of the wave. An additional study of the massive multi-input multi-output radar is deeply placed where components are well-positioned, which produces strength to the spatial distinctiveness [29,30]. The colocated scanned massive radar brings a very good spatial intent parameter point of the target recognition analysis if we tried to compare it with wide-spaced radar [31]. Consider x(t) to be the signal transmitted by M massive multi-input multi-output radar input presented here,

(1) $$x\left(t\right)={\left[x_{1}\left(t\right)e^{Jw_{c}t}x_{2}\left(t\right)e^{Jw_{c}t}\ldots x_{M}\left(t\right)e^{Jw_{c}t}\right]}^{T}$$

where $x_{k}\left(t\right)e^{Jw_{c}t}$ represents non-modulated signal band of $k^{th}$ transmitted radar antenna with $\omega_{c}$ which represent the frequency jagged or redirected in Hertz, t ∈ [0,$T_{0}]$, with $T_{0}$ being the observation time. We define the transmit steering vector as,

(2) $$a_{T}\left(\theta \right)={\left[e^{-Jw_{c}\tau T_{1}\left(\theta \right)}e^{-Jw_{c}\tau T_{2}\left(\theta \right)}\ldots e^{-Jw_{c}\tau T_{M}\left(\theta \right)}\right]}^{T}$$

Then, the transmit-receive steering matrix can be written as,

(3) $$A\left(\theta \right)≜a_{R}\left(\theta \right)a_{T}^{T}$$

By considering *M* transmitter and receiver, we can then define,${\;a}_{T}\left(\theta \right)≜a\left(\theta \right)≜a_{R}\left(\theta \right)$.

For flexible reasoning, we assume that the path attenuation of the wave α is the same for the transmitter and receivers’ antennas; we use this inference because of the backside site [32]. The tilt *θ* represents the angular azimuth of the chosen point. A summary of notations presented in this paper can be found in Table 1. The signal received from a single point, in far-end with constant velocity $\upsilon_{r}$ at an angle *θ* can be written as,

(4) $$y\left(t\right)=\alpha A\left(\theta \right)x\left(t\right)+n\left(t\right)$$

where *α* is the loss path as well as the breeding loss and the reflection measurement, and $n\left(t\right)$ represents additive complex Gaussian noise. On the receiver side, we set the following inference:

−θ and α are deterministic unknown parameters and is the entrance orientation of the chosen target and complex magnitude of the target, respectively. We denote the move of trajectory noise by $n\left(t\right)$, and is independent, zero-mean, we consider it to be well-known, complex Gaussian and converged matrix, $R_{n}=\sigma_{n}^{2}I_{M}$, i.e., $n\left(t\right)$~ ${\mathbb{N}}^{c}$($0_{M}$,${\;\sigma }_{n}^{2}I_{M}$), where ${\mathbb{N}}^{c}$ represents the CGND (Complex Gaussian Normally Distributed). By Considering Equation (4) hypothesis, the receiver signal can be represented,

(5) $$y(t)\sim {\mathbb{N}}^{c}\left(\alpha A\left(\theta \right)x\left(t\right),\sigma_{n}^{2}I_{M}\right)$$

And the orthogonal waveforms transmitted by the massive MIMO radars can be written,

(6) $$R_{x}=\int_{T_{0}}X\left(t\right)X^{H}\cdot dt=I_{M}$$

The quadratic transmission signals of massive MIMO radars advantages in context of selecting one specific receiver from the transmission side and generate end-to-end inclusion system to ameliorate the angle of resolution, increase the cluster hole more on virtualization, also enlarge the number of solvable targets, reduces earlobes [33], and decrease the probability of head off if we tried to compare it to the rational signal waves [34].

#### 2.1.1. Mobile Communication System

A TDD massive multi-input multi-output mobile LTE communications system has been considered for Ҡ base stations; each is supplied by an $N^{\mathrm{BS}}$ transmitter and receiver element, shielded by $i^{th}$ base station and $L_{i}^{\mathrm{UE}}$ user equipment. The user equipment likewise are multi-input and output systems categorized by $N^{\mathrm{UE}}$ transmitter and receiver element. By conceding that $s_{i}^{\mathrm{UE}}$(t) is the transmitted signal of $j^{th}$ user equipment to the $i^{th}$ unit, the receiver signal at the end base station can be described,

(7) $$r_{i}\left(t\right)=\sum_{j}^{\;}Q_{c,j}H_{j}^{N^{\mathrm{BS}}x\;{\mathbb{N}}^{\mathrm{UE}}\;}P_{c,j}s_{i}^{\mathrm{UE}}\left(t\right)+Q_{c,j}w_{j}\left(t\right)\;1\leq j\leq L_{i}^{\mathrm{UE}}$$

where $H_{j}\;$represents the matrix in $j^{th}$ user equipment communication system, $w_{j}\left(t\right)$ represents the white additive Gaussian noise. $Q_{c,j}$,${\;P}_{c,j}$ represents the linear decoding and precoding matrices. The goal of designing the $j^{th}$ precoder and decoder is to find a null space spanned by the columns of a decoder matrix in order to align the interfering signals [11,35,36,37].

#### 2.1.2. Co-Existence RF Environment

In communication wireless system model theory, we generally assumed that the transmission (which is from the base station) carried the States Space Channel Information (SSCI) from the receiver (known as the user equipment) in Frequency Division Duplex communication systems. On the other hand, they can exchange each other’s transmission channel in Time Division Duplex communication systems [38]. Response and exchange traffic are well-grounded, feasible as much as the response has an understandable and consistent time and radio frequency channel and greater than the reciprocity traffic time, respectively. For instance, when radar channels are sharing spectrum with mobile cellular structure, one way for getting the SSCI is that the radar shall measure $H_{i}\;$according to the estimation sent from the base station (BS) [39]. A different method is that the radar gets the advantage of mobile cell concerning carrier estimation, assisted by the low-power signal, and the carrier estimation is filtered to the radar. For instance, by considering radar detected signal as an interference on the mobile cellular side, the channel can be classified as an intrusion channel and consider the internal information as intrusion channel state information. The propagation of the spectrum between radar and LTE mobile cellular system networks can be visualized in two main ways, first as the radars’ military network system. Splitting their spectrum within military base stations, we denote it Mil-to-Mil spectrum sharing. The second way is the radars’ military base station sharing their spectrum with a business or mobile cellular commercial network. This was denoted as the Mil-to-Com spectrum sharing. In this paper, we focused more on the Mil-to-Com case. The intrusion channel state information can be obtained by allowing impulse of commercial network. The largest impulse scheme is the null-pilot and shelter that came from the radar interference. In the two scenarios, notwithstanding the sharing scheme, Mil-to-Mil or Mil-to-Com, we have the intrusion channel state information for the reason of reducing radar interference at the mobile commercial network. 

#### 2.1.3. Construction

**Figure 1:**
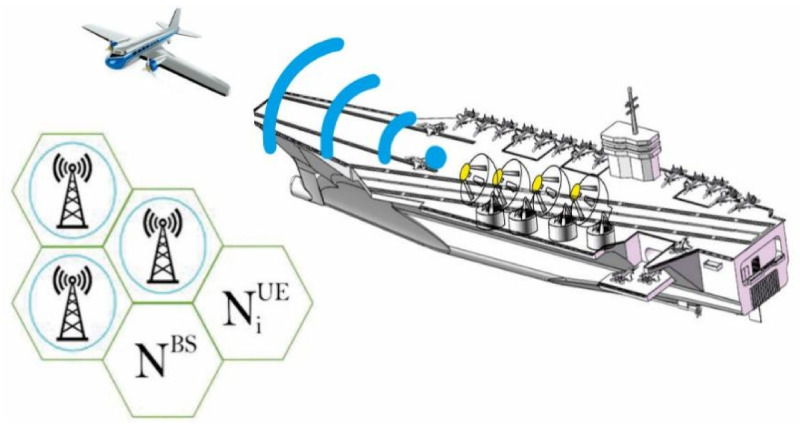
Spectrum-sharing description for massive MIMO radar and target point at the same time splitting spectrum with massive MIMO mobile cellular network.

We harmonized the coexistence between the two schemes as illustrated in Figure 1, in which the military-based massive radar multi-input multi-output is splitting Ҡ interference carriers with its neighbor mobile system. By considering this scenario, the detected signal on the receiver side at the $i^{th}$ base station can be represented as,

(8) $$r_{i}\left(t\right)=H_{i}^{N^{\mathrm{BS}}x\;M\;}x\left(t\right)+\sum_{j}^{\;}Q_{c,j}H_{j}^{N^{\mathrm{BS}}x\;{\mathbb{N}}^{\mathrm{UE}}\;}P_{c,j}s_{i}^{\mathrm{UE}}\left(t\right)+Q_{c,j}w_{j}\left(t\right)$$

$H_{i}$ represents interference channel between mobile Cellular base station and the radar. And i = 1,2,…, k, where $H_{i}$ can be written,

(9) $$H_{i}=\left[\begin{array}{ccc} h_{i}^{\left(1,1\right)} & \cdots & h_{i}^{\left(1,M\right)}\\ \vdots & \ddots & \vdots \\ h_{i}^{(N^{\mathrm{BS}},\;1)\;} & \cdots & h_{i}^{\left(N^{\mathrm{BS}},M\;\right)} \end{array}\right]\left(N^{BS}\times M\right)$$

Here $h_{i}^{\left(l,k\right)}$ denotes the coefficient carrier of $k^{th}$ radar’s base station antenna to the $l^{th}$ LTE mobile antenna of the $i^{th}$ base station. The components of $H_{i}$ are independent, identically distributed, moreover annular proportional and randomly distributed equivalent to complex Gaussian with zero-mean, hence accepting Rayleigh dispersion. Furthermore, meticulous and comprehensive analysis of interference channel for radar and mobile communications systems, along with more than two special channels, are presented in [28,33,40]. Our goal here is to map x(t) toward null space interference $H_{i}$, by canceling interference at $i^{th}$ base station, such as $H_{i}x\left(t\right)=0$, so that $r_{i}\left(t\right)$ should be Equation (7) instead of Equation (8).

### 2.2. Radar Mobile System Spectrum Sharing

In this section, we deal with spectrum sharing of radar multi-input multi-output and that of mobile communication, and included Ҡ base stations. Both systems share the same numbers of interferences (Ҡ) that lead us to $H_{i}$ (i = 1, 2, …, k). The detected signal of radar is estimated by projecting it to the map of zero interference channel and connecting the two communications systems (radar and mobile) by utilizing our suggested interference-channel-collection inference, in sequence of having removed all interferences from the radar multi-input multi-output. The selection of interference channel is done with respect on maximizing zero map projection, represented as $\underset{1\leq i\leq Ҡ}{\mathrm{argmax}}$ $dim\left[\mathbb{N}\left(H_{i}\right)\right]$ and project the detected signal of the radar in null space of this scheme. 

#### 2.2.1. Performance

We used theorem of Cramer bound and the maximum likelihood estimation to evaluate the slope of the targeted point of entrance as our statistical scheme of the network. Attention also was put on analyzing the deterioration approximation of the arrival angle of the chosen point, suitable to project the wave of the radar in null space. Cramer bound of an isolated chosen point study was well analyzed in [40],

(10) $$\mathrm{CB}=\frac{1\;}{\;2\;\mathrm{SNR}}{\left(M_{R}\dot{a_{T}^{H}}\left(\theta \right)R_{x}^{T}\dot{a_{T}}\left(\theta \right)+a_{T}^{H}\left(\theta \right)R_{x}^{T}a_{T}\left(\theta \right)\|\dot{a_{R}}{\left(\theta \right)\|}^{2}-\frac{M_{R}{\left|a_{T}^{H}\left(\theta \right)\left.R_{x}^{T}\dot{a_{T}}\left(\theta \right)\right|\right.}^{2}}{a_{T}^{H}\left(\theta \right)R_{x}^{T}a_{T}\left(\theta \right)}\right)}^{-1}$$

For instance, the maximum likelihood of no interference of a single targeted point can be represented as,

(11) $${\left(\hat{\theta },\hat{\tau },{\hat{\omega }}_{D}\right)}_{ML}=\underset{\theta ,\tau ,\omega_{D}}{\mathrm{argmax}}\frac{{\left|a_{T}^{H}\left(\theta \right)\left.E\left(\tau_{r},\omega_{D}\right)\dot{a_{T}^{*}}\left(\theta \right)\right|\right.}^{2}}{M_{R}a_{T}^{H}\left(\theta \right)R_{x}^{T}a_{T}\left(\theta \right)}$$

where,

$$\begin{array}{c} \dot{a_{R}}\left(\theta \right)=\frac{da_{R}\left(\theta \right)}{d\theta }\\ \dot{a_{T}}\left(\theta \right)=\frac{da_{T}\left(\theta \right)}{d\theta }\\ R_{x}=\int_{T_{0}}X\left(t\right)X^{H}\left(t\right)\cdot dt\\ E\left(\tau_{r},\omega_{D}\right)=\int_{T_{0}}y\left(t\right)X^{H}\left(t-\tau_{r}\right)\cdot dt \end{array}$$

where $\tau_{r}$ is the two-way breeding hold up connecting the chosen point and the reference point, and we denote $\omega_{D}$ as the Doppler frequency transfer. Furthermore, we consider the performance measurement such as the Cramer bound and maximum likelihood; we are more concerned with the constant changes that occurred in the beampattern of radar wave projection. The response of beampattern measurement for a chosen point is controlled by *θ* as presented in [40],

(12) $$G\left(\theta ,\theta_{D}\right)=\Omega \frac{{\left|a_{T}^{H}\left(\theta \right)\left.R_{x}^{T}a_{T}\left(\theta_{D}\right)\right|\right.}^{2}}{a_{T}^{H}\left(\theta_{D}\right)R_{x}^{T}a_{T}\left(\theta_{D}\right)}\frac{{\left|a_{R}^{H}\left(\theta \right)\left.a_{R}\left(\theta_{D}\right)\right|\right.}^{2}}{M_{R}}$$

Here Ω represents the constant of harmonization, and $\theta_{D}$ is the processor-driving rudder of the primary beam. In this research, we studied two spectrum-sharing methods and analyzed them as follows:

**Case 1 (*M***<**Ҡ**$N^{BS}$**and*****M >***$N^{BS}$**)**. A case where the radar has only a few beam antennas in comparison with the interconnected, which is Ҡ-BS where *M* <Ҡ$N^{BS}$. On the other hand, radar antennas are greater than the base station antennas beam, where *M >* $N^{BS}$. In a situation like this, it is impossible for the radar to reduce interferences at once in all the antennas as Ҡ-Base station inside the system structure are due to poor applicable degrees of freedom. Moreover, the accessible degrees of freedom can grant us target detection and interference reduction at once, and that means only upon one selected base station inside the network of Ҡ base stations. For the selection of the base station by the radar, this will depend on the optimization operation of the radar. This paper seeks to study how to reduce interference in a maximum manner on the side of the mobile base station with the least possible deterioration performance of the radar operation. The defect is that the interferences cannot significantly be reduced by removing one base station on the network; therefore, the radar will have to use a very high-power level in order to have a good performance. However, this can increase the probability gain of interference on the mobile base stations which are not included in the mitigation study scenario. In [41,42], the technique explained that by applying resource allocation and dual-cell approaches we can change Ҡ – 1 base stations to nonradar frequency ranges. In M <Ҡ$N^{BS}$ conventional colocated multi-input multi-output radar and cellular system, applying the Zero Interference Projection (ZIP) technique, is not an effective way of reducing significant interference because ZIP has a limited number of parameters to stabilize the two systems. By so doing, it will lead to low performance of the radar. Although we can also modify the structure of the radar system into a superposed multi-input multi-output radar structure, the transmitted wave range of colocated radar will have to be divided into numerous sub-rages which can be acceptable by superposed. A superposed radar waves structure increases the degree of freedom of the transmitted wave, and as result the massive multi-input multi-output radar can perform very well. At the same time, we have significantly reduced interferences at the LTE mobile base station without compromising the radar’s transmitted parameters requirement.

**Case 2Where *M >***$ҠN^{BS}$. Let us examine a case where the massive multi-input multi-output radar has many antenna elements and this increases the beam, in comparison with a mixed antenna element with Ҡ base stations. In such a situation, it is possible that the massive multi-input multi-output radar can reduce considerable interference to every single K base station in the network, while ensuring good signal detection of the chosen targets. Here we have enough degree of freedom which makes our scenario possible. In this case, the mixed interference shared in the networks between massive multi-input multi-output radar and LTE mobile base stations system can be written as;

(13) $$H=\left[H_{1},H_{2}\ldots H_{Ҡ}\right]$$

#### 2.2.2. Matrix Projection

At this point, we structure the prediction array for Scenarios 1 and 2.

1st Prediction Scenario, where *M* <Ҡ$N^{BS}$ and *M >* $N^{BS}$: Here, the prediction algorithm for the first scenario is presented. At this point, the schemes of radar detection are estimated over null space of interference transmission trajectory $H_{i}$. We suppose that the MIMO radar has the information of each channel state of $H_{i}$ interference channels, over the response, in Mil-to-Mil or Mil-to-Com scheme, considering the performance of a unique evaluation of decomposition (UED) to estimate the interference cancellation and therefore build the projection matrix. Let us estimate first EUD of $H_{i}$,

(14) $$H_{i}=U_{i}\;\Sigma_{i}\;V_{i}^{H}$$

And,

(15) $$\tilde{\Sigma_{i}}≜diag\left(\tilde{\sigma_{i1}},\tilde{\sigma_{i2}},\ldots ,\tilde{\sigma_{ip}}\right)$$

where *p*≜ min ($N^{BS},M$) and $\tilde{\sigma_{i1}}>\tilde{\sigma_{i2}}>\cdots >\tilde{\sigma_{iq}}>\tilde{\sigma_{iq+1}}$=$\tilde{\sigma_{iq+2}}=\cdots =\tilde{\sigma_{ip}}=0$ are the singular values of $H_{i}$. From here we can define,

(16) $${\tilde{\Sigma_{i}}}^{\prime }≜diag\left({\tilde{\sigma_{i1}}}^{\prime },{\tilde{\sigma_{i2}}}^{\prime },\ldots ,{\tilde{\sigma_{iM}}}^{\prime }\right)$$

where,

$${\tilde{\sigma_{i,u}}}^{\prime }≜\left\{\begin{array}{c} \;0\;,\forall \;u\leq q\;,\;\\ 1,\forall \;u>q, \end{array}\right.$$

Given aforementioned definitions, we are able to determine projection matrix,

(17) $$P_{i}≜V_{i}\;{\tilde{\Sigma_{i}}}^{\prime }\;V_{i}^{H}$$

In order to verify that $P_{i}$ is a suitable matrix projection, these two conditions have to be met:

Condition 1 $P_{i}$ ∈ ${ℭ}^{M\times M}$ is an appropriate projection matrix if, ∀ $P_{i}$ ⇔ $P_{i}^{H}$=$P_{i}^{2}$,

Proof, $P_{i}$ = $P_{i}^{H}$, considering Hermitian of Equation (17) we have,

(18) $$P_{i}^{H}={\left(V_{i}\;{\tilde{\Sigma_{i}}}^{\prime }\;V_{i}^{H}\right)}^{H}=P_{i}$$

(19) $$P_{i}^{2}=V_{i}\;\tilde{\Sigma_{i}}\;V_{i}^{H}\times V_{i}\;\tilde{\Sigma_{i}}\;V_{i}^{H}=P_{i}$$

$V_{i}\;V_{i}^{H}=I$, here it is form orthogonal matrices where ${\left({\tilde{\Sigma_{i}}}^{\prime }\right)}^{2}={\tilde{\Sigma_{i}}}^{\prime }$. Based on Equation (18) as well as (19) is follows that, $P_{i}$ = $P_{i}^{H}$ = $P_{i}^{2}$. We can also show $P_{i}$ is a projector matrix if ∀ v ∈ rang ($P_{i}$), then $P_{i}v=v;\;and\;w,v=P_{i}w$, then,

$$P_{i}v=P_{i}\left(P_{i}w\right)=P_{i}{}^{2}w=P_{i}w=v$$

Moreover, $P_{i}v-v\in null\left(P_{i}\right),\;and$

$$P_{i}\left(P_{i}v-v\right)=P_{i}{}^{2}v-P_{i}v=P_{i}v-P_{i}v=0$$

This confirms our first property. 

Condition 2 $P_{i}$ ∈ ${ℭ}^{M\times M}$ is the null space orthogonally projected matrix $H_{i}\in {ℭ}^{N^{BS}\times M}$.

Proof, $P_{i}$ = $P_{i}^{H}$,we can write,

(20) $$H_{i}P_{i}^{H}=U_{i}\tilde{\Sigma_{i}}V_{i}^{H}\times V_{i}\;{\tilde{\Sigma_{i}}}^{\prime }\;V_{i}^{H}=0$$

$\tilde{\Sigma_{i}}{\tilde{\Sigma_{i}}}^{\prime }=0$ (by structure).

In ‘Prediction Scenario 1′, we have a total of Ҡ interference channels. Hence, we must choose the channel that leads to a minimum deterioration of radar wave in the lowest level as possible,

(21) $$i_{min}≜\;\arg\underset{1\leq i\leq Ҡ}{\max}\begin{array}{c} {\left|\left|P_{i}x\left(t\right)-x\left(t\right)\right|\right|}_{2} \end{array}$$

$$P_{i_{min}}≜\overset{ˇ}{P}$$

After the projection matrix has been determined, we can now estimate the signal of radar over the null space of intrusion channel,

(22) $$\overset{ˇ}{x}\left(t\right)=\overset{ˇ}{P}x\left(t\right)$$

The statistical matrix between the two waves can be written as,

(23) $$R_{\overset{ˇ}{x}}=\int_{T_{0}}\overset{ˇ}{x}\left(t\right){\overset{ˇ}{x}}^{H}\left(t\right)\cdot dt$$

The projection does not preserve its perpendicularity, which means it is not identical anymore and is classified according to the projection matrix.

2nd Prediction Scenario, where *M >* $ҠN^{BS}$: Here, the prediction algorithm for the second scenario is presented. At this point, the schemes of radar detection are estimated over null space of mixed interference trajectory H. It can be written as,

(24) $$H=U\;\Sigma \;V^{H}$$

And,

(25) $$\tilde{\Sigma }≜diag\left(\tilde{\sigma_{1}},\tilde{\sigma_{2}},\ldots ,\tilde{\sigma_{p}}\right)$$

where *p*≜ min ($N^{BS},M$) and $\tilde{\sigma_{1}}>\tilde{\sigma_{2}}>\cdots >\tilde{\sigma_{q}}>\tilde{\sigma_{q+1}}$=$\tilde{\sigma_{q+2}}=\cdots =\tilde{\sigma_{p}}=0$ are the singular values of H. We define,

(26) $${\tilde{\Sigma_{i}}}^{\prime }≜diag\left({\tilde{\sigma_{1}}}^{\prime },{\tilde{\sigma_{2}}}^{\prime },\ldots ,{\tilde{\sigma_{M}}}^{\prime }\right)$$

where,

$${\tilde{\sigma_{u}}}^{\prime }≜\left\{\begin{array}{c} \;0\;,\forall \;u\leq q\;,\;\\ 1,\forall \;u>q, \end{array}\right.$$

Considering the present assumption, we can then extend the projection matrix as follows,

(27) $$P≜V\;{\tilde{\Sigma }}^{\prime }\;V^{H}$$

At this point we can now conclude that P is an accurate projection matrix based on Conditions 1 and 2.

### 2.3. Target Detection

In this point, we expended a numerical detection test to improve the decision with respect to orthogonal wave of radar, including Null-space projection waves. Our objective here is a comparative test scheme of the waveforms through analyzing decision test statistics, that is, if the targeted point is present or not in the scope of Doppler shift needed. By considering the target detection and evaluation we analyzed by considering the hypothesis estimation test, wherefrom we decide to pick between two hypotheses: the zero hypothesis ${Ԣ}_{0}$ associating the case where the target is absent, and hypothesis ${Ԣ}_{1}$ associating the case where the target is present. For one model, the hypothesis test as in Equation (4) can be presented as,

(28) $$y\left(t\right)=\left\{\begin{array}{c} \;n\left(t\right)\;\;\;\;\;\;\;\;\;\;\;\;\;\;\;\;\;\;\;\;\;\;\;\;\;\;\;\;:{Ԣ}_{0\;}\;\left(\mathrm{Absent}\right)\;\;0\leq \;t\leq \;T_{0\;}\\ \alpha A\left(\theta \right)x\left(t\right)+n\left(t\right)\;:{Ԣ}_{1}\;\left(\mathrm{Present}\right)\;0\leq \;t\leq \;T_{0\;} \end{array}\right.$$

We used the generalized likelihood ration test because α and θ are unknown parameters but deterministic. The benefit of utilizing the generalized likelihood ration test is that we can substitute the hidden parameters with their maximum likelihood computation. The maximum likelihood estimations of α and θ can be found for different signal schemes, point targets, and noise sources here in [40,43] where the orthogonal signals are used. In this paper, we examine a system with one target, and we did not consider the source of interference so we can better analyze the effect of NSP on target sensing. Consequently, we put forward an easier method of the maximum likelihood and generalized likelihood ration test estimation. The detection scheme of Equation (4) can then be represented,

(29) $$y\left(t\right)=Q\left(t,\theta \right)\alpha +n\left(t\right)$$

And,

(30) $$Q\left(t,\theta \right)=A\left(\theta \right)x\left(t\right)$$

We take the advantage of Karhunen–Loève algorithm to derive the log-likelihood function on evaluating α and θ. We consider Ω to be the set, where elements are {y(t),Q(t, θ),n(t)}. We denote $\psi_{z}$, z = 1, 2, …, are the orthonormal function of Ω meeting the requirement,

$$\langle \psi_{z}\left(t\right),\psi_{z^{\prime }}\left(t\right)\rangle =\int_{T_{0}}\psi_{z}\left(t\right),\psi_{z^{\prime }}^{*}\left(t\right)=\delta_{{\mathrm{zz}}^{\prime }}$$

where $\delta_{{\mathrm{zz}}^{\prime }}$ represents a function of Krönecker. This next series of elements of Ω, can be expressed to develop the system, y(t), Q(t, θ)}, and n(t),

(31) $$y\left(t\right)=\overset{\infty }{\underset{z=1}{\sum }}y_{z}\psi_{z}\left(t\right)$$

(32) $$Q\left(t,\theta \right)=\overset{\infty }{\underset{z=1}{\sum }}Q_{z}\left(\theta \right)\psi_{z}\left(t\right)n\left(t\right)=\overset{\infty }{\underset{z=1}{\sum }}n_{z}\psi_{z}\left(t\right)$$

Here $y_{z}$,${\;Q}_{z},$ and $n_{z}$ repesent weighed rate of Karhunen–Loève estimation in the view of the process gained by considering the matching internal production through the basic function $\psi_{z}\left(t\right)$. Therefore, a corresponding discrete scheme of Equation (29) can be written as,

(33) $$y_{z}=Q_{z}\left(\theta \right)\alpha +n_{z},\;z=1,2$$

Considering the white annular complex Gaussian can be represented as:

Ḓ[n(t)nt-nτ(t)] = $\sigma_{n}^{2}I_{M}\;\delta (\tau t$), [2] the array is i.i.d and $n_{z}$~ ${\mathbb{N}}^{c}$($0_{M}$,${\;\sigma }_{n}^{2}I_{M}$). From here, the function of log-likelihood can be expressed,

(34) $$L_{y}\left(\theta ,\alpha \right)=\overset{\infty }{\underset{z=1}{\sum }}\left(-\mathrm{Mlog}\left({\pi \sigma }_{n}^{2}\right)-\frac{1}{\sigma_{n}^{2}}{\left|\left|y_{z}-Q_{z}\left(\theta \right)\alpha \right|\right|}^{2}\right)$$

By Maximizing Equation (34),

(35) $$L_{y}\left(\theta ,\hat{\alpha }\right)=\Gamma -\frac{1}{\sigma_{n}^{2}}\left(E_{yy}-e_{Qy}^{H}E_{QQ}^{-1}e_{Qy}\right)$$

where,

$$\begin{array}{c} \Gamma ≜-Mlog\left(\pi \sigma_{n}^{2}\right)\\ E_{yy}≜\overset{\infty }{\underset{z=1}{\sum }}{\left|\left|y_{z}\right|\right|}^{2}\Rightarrow \int_{T_{0}}{\left|\left|y_{z}\right|\right|}^{2}dt\\ e_{Qy}≜\overset{\infty }{\underset{z=1}{\sum }}Q_{z}^{H}y_{z}\Rightarrow \int_{T_{0}}Q^{H}\left(t,\theta \right)y\left(t\right)dt\\ E_{QQ}^{-1}≜\overset{\infty }{\underset{z=1}{\sum }}Q_{z}^{H}Q_{z}\Rightarrow E_{QQ}=\int_{T_{0}}Q^{H}\left(t,\theta \right)Q\left(t,\theta \right)dt \end{array}$$

It must be noted that Equation (35), aside of the constant Γ, the other additions lead to infinite. However, because of the noncontribution of highest rank condition, the evaluation of *θ* and *α* their total sum can be finite by applying the equality,

$$\int_{T_{0}}V_{1}\left(t\right)V_{2}^{H}\left(t\right)dt=\overset{\infty }{\underset{z=1}{\sum }}V_{1z}V_{2z}^{H}$$

And $V_{i}\left(t\right)=$ $\overset{\infty }{\underset{z=1}{\sum }}V_{1z}\psi_{z}\left(t\right)$, *i* = 1, 2. We can describe $f^{\mathrm{th}}$ element of $e_{\mathrm{Qy}}$, after bringing Q (t, *θ*) to Equation (30).

(36) $${\left[e_{Qy}\right]}_{f}=a^{H}\left(\theta_{f}\right)E^{T}a\left(\theta_{f}\right)$$

where,$\;E=\int_{T_{0}}y\left(t\right)x^{H}\left(t\right)dt$, the same way, we can describe f $g^{\mathrm{th}}$ element of $E_{QQ}$ written here,

(37) $${\left[E_{QQ}\right]}_{fg}=a^{H}\left(\theta_{f}\right)a\left(\theta_{g}\right)a^{H}\left(\theta_{f}\right)R_{x}^{T}a\left(\theta_{g}\right)$$

Since, $e_{Qy}$ and $E_{QQ}$ does not depend on the receiver, then the statistical estimation of *θ* and α can be taken from Equations (36) and (37). Now the maximum log-likelihood function can be represented as a vector estimation,

$$L_{y}\left({\hat{\theta }}_{ML}\right)=\;\arg\underset{\theta }{\max}\frac{{\left|a^{H}\left({\hat{\theta }}_{ML}\right)Ea^{*}\left({\hat{\theta }}_{ML}\right)\right|}^{2}}{Ma^{H}\left({\hat{\theta }}_{ML}\right)R_{x}^{T}a\left({\hat{\theta }}_{ML}\right)}$$

And our hypothesis testing model as in Equation (28), it becomes then,

(38) $$L_{y}=\underset{\theta ,\alpha }{\max}\left\{\log f_{y}\left(y,\theta ,\alpha ;{Ԣ}_{1}\right)\right\}-logf\left(y;{Ԣ}_{0\;}\right)\begin{array}{cc} \begin{array}{c} {Ԣ}_{0\;}\\ ≶\\ {Ԣ}_{1}\; \end{array} & \delta \end{array}$$

where $f_{y}\left(y,\theta ,\alpha ;{Ԣ}_{1}\right)$ and $f\left(y;{Ԣ}_{0\;}\right)$ are the result of the hypothesis test ${Ԣ}_{0\;}$and ${Ԣ}_{1}$ respectively, which is the probability density functions of the receiver. Hence, the generalized likelihood ration test can be expressed as,

(39) $$L_{y}\left({\hat{\theta }}_{ML}\right)=\arg\underset{\theta }{\max}\frac{{\left|a^{H}\left({\hat{\theta }}_{ML}\right)Ea^{*}\left({\hat{\theta }}_{ML}\right)\right|}^{2}}{Ma^{H}\left({\hat{\theta }}_{ML}\right)R_{x}^{T}a\left({\hat{\theta }}_{ML}\right)}\begin{array}{cc} \begin{array}{c} {Ԣ}_{0\;}\\ ≶\\ {Ԣ}_{1}\; \end{array} & \delta \end{array}$$

The statistic of $L\left({\hat{\theta }}_{ML}\right)$ for both the hypotheses can be found in [44].

(40) $$L\left({\hat{\theta }}_{ML}\right)\sim \left\{\begin{array}{c} \;{Ԣ}_{0\;}:X_{2}^{2}\\ {Ԣ}_{1}:X_{2}^{2}\left(\rho \right) \end{array}\right.$$

Whereby,

— $X_{2}^{2}\left(\rho \right)$ represents the chi-squared noncentral dispensations, having 2 as degrees of freedom (DoF),
— $X_{2}^{2}$ represents centralized chi-squared dispensations, having 2 as degrees of freedom (DoF). ρ represents the noncentral parameter; it can be written as,
  $$\rho =\frac{\alpha^{2}}{\sigma_{n}^{2}}{\left|a^{H}\left(\theta \right)R_{x}^{T}a\left(\theta \right)\right|}^{2}$$

Based on a chosen probability of false alarm, for a given signal detection, a ration δ must be generated,

(41) $$P_{f}=Pr\left[L\left(y\right)>\delta \backslash {Ԣ}_{0\;}\right]$$

$$\delta =F_{X_{2}^{2}}^{-1}\left(1-P_{f}\right)$$

Whereby $F_{X_{2}^{2}}^{-1}$ represents the inverse dispensations function with 2 as DoF. Then the signal detection estimation can be presented as,

(42) $$\begin{array}{c} P_{D}=\mathrm{Pr}\left[L\left(y\right)>\delta \backslash {Ԣ}_{1}\right]\\ P_{D}=1-F_{X_{2}^{2}\left(\rho \right)}\left[F_{X_{2}^{2}}^{-1}\left(1-P_{f}\right)\right] \end{array}$$

Whereby $F_{X_{2}^{2}\left(\rho \right)}$ is the noncentral dispensations function including its parameter ρ.

#### 2.3.1. P_D_ for Orthogonal Waveforms

For orthogonal waveforms $R_{x}^{T}=I_{M}$, therefore, the generalized likelihood ration test can be expressed as,

$$L_{O}\left({\hat{\theta }}_{ML}\right)=\frac{{\left|a^{H}\left({\hat{\theta }}_{ML}\right)Ea^{*}\left({\hat{\theta }}_{ML}\right)\right|}^{2}}{Ma^{H}\left({\hat{\theta }}_{ML}\right)a\left({\hat{\theta }}_{ML}\right)}\begin{array}{cc} \begin{array}{c} {Ԣ}_{0\;}\\ ≶\\ {Ԣ}_{1}\; \end{array} & \delta_{O} \end{array}$$

and the estimation of $L\left({\hat{\theta }}_{ML}\right)$ for this scheme can be written,

$$L_{O}\left({\hat{\theta }}_{ML}\right)\sim \left\{\begin{array}{c} \;{Ԣ}_{0\;}:X_{2}^{2}\\ {Ԣ}_{1}:X_{2}^{2}\left(\rho_{O}\right) \end{array}\right.$$

where,

$$\rho_{O}=\frac{M^{2}{\left|\alpha \right|}^{2}}{\sigma_{n}^{2}}$$

$\delta_{O}$ is defined following a required probability of false alarm,

$$\delta_{O}=F_{X_{2}^{2}}^{-1}\left(1-P_{f-Ort}\right)$$

And then we can determine the detection for orthogonal waves as follows,

(43) $$P_{D-Ort}=1-F_{X_{2}^{2}\left(\rho_{O}\right)}\left[F_{X_{2}^{2}}^{-1}\left(1-P_{f-Ort}\right)\right]$$

#### 2.3.2. P_D_ for NSP Waveforms

For spectrum-sharing waveforms $R_{x}^{T}=R_{\overset{ˇ}{x}}^{T}$, therefore, the generalized likelihood ration test can be expressed as,

$$L_{NSP}\left({\hat{\theta }}_{ML}\right)=\frac{{\left|a^{H}\left({\hat{\theta }}_{ML}\right)Ea^{*}\left({\hat{\theta }}_{ML}\right)\right|}^{2}}{Ma^{H}\left({\hat{\theta }}_{ML}\right)R_{\overset{ˇ}{x}}^{T}a\left({\hat{\theta }}_{ML}\right)}\begin{array}{cc} \begin{array}{c} {Ԣ}_{0\;}\\ ≶\\ {Ԣ}_{1}\; \end{array} & \delta_{NSP} \end{array}$$

and the estimation of $L\left({\hat{\theta }}_{ML}\right)$ for this scheme can be written, 

Here,

$$L_{NSP}\left({\hat{\theta }}_{ML}\right)\sim \left\{\begin{array}{c} \;{Ԣ}_{0\;}:X_{2}^{2}\\ {Ԣ}_{1}:X_{2}^{2}\left(\rho_{NSP}\right) \end{array}\right.$$

$$\rho_{NSP}=\frac{\alpha^{2}}{\sigma_{n}^{2}}{\left|a^{H}\left(\theta \right)R_{\overset{ˇ}{x}}^{T}a\left(\theta \right)\right|}^{2}$$

$\delta_{NSP}$ is also defined following a required probability of false alarm,

$$\delta_{O}=F_{X_{2}^{2}}^{-1}\left(1-P_{f-Ort}\right)$$

And we can then determine the detection for orthogonal waves as follows,

(44) $$P_{D-NSP}=1-F_{X_{2}^{2}\left(\rho_{NSP}\right)}\left[F_{X_{2}^{2}}^{-1}\left(1-P_{f-NSP}\right)\right]$$

## 3. Numerical Results

For better analysis of the detection sequence execution of each spectrum propagation for the massive multi-input multi-output radars, the Monte Carlo execution was performed by simulation on manipulating radar’s parameter as presented in [45]. 

### 3.1. Analysis of Scenario 1

For this scenario, we generated Ҡ Rayleigh channels interference at every run of the Monte Carlo simulation. We have the dimensions $N^{\mathrm{BS}}\times M$, and computed the null spaces and built matching projection matrix by applying Algorithm 2. We decided the finest channel to carry out projection of radar signal scheme on applying Algorithm 1, transmit Null Space Projection signal scheme based on the received signal detection. We calculated the parameters estimation of θ, α, and estimated the detection signal sequence for orthogonal and NSP waves.

**Figure 2:**
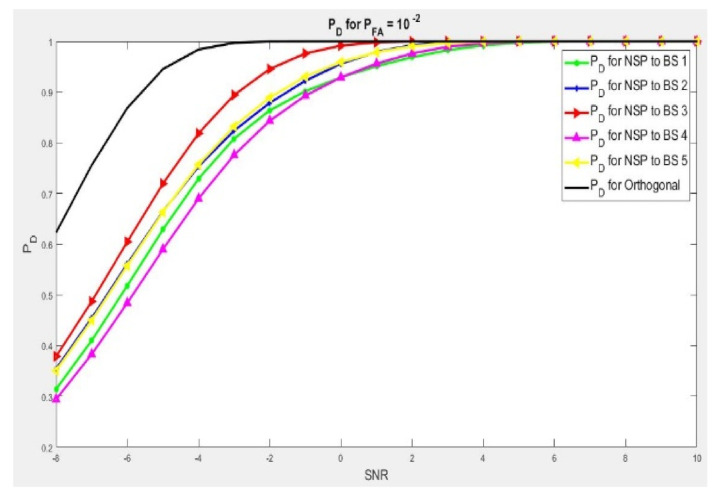
$P_{D}$ when dimension $\mathbb{N}\left(H_{i}\right)$ = 1. And 3 to 6 dB more gain in NSR is needed to achieve 90% of target detection.

**Figure 3:**
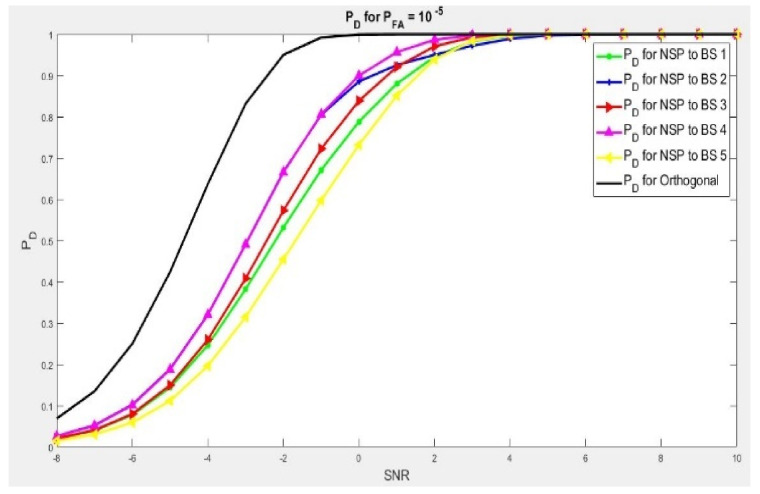
$P_{D}$ when dimension $\mathbb{N}\left(H_{i}\right)$ = 6. And 2.3 to 2.6 dB is needed to achieve 90% of target detection.

We performed Algorithm 1 and 2. In Figure 2 and Figure 3, we demonstrate the importance of the two algorithms (1 and 2), in enhancing the detection target point where many BSs are in use on the detection scheme of the radar. It has to correctly detect the target point while not disturbing the sensing signal environment of the mobile LTE system scheme concerned. A summary of test environment parameters are presented in Table 2. For instance, we examined the case with five base stations (BSs) and the radar will have to choose the best projection channel with a minimum degradation scheme waveform within, while consequently maximizing the contingency of target detection.

In the scenario with $\mathbb{N}\left(H_{i}\right)$ = 1 as shown in Figure 2, we presented results of five different Null Space Projection detection signals. This means that radar’s waves are projected with five base station signals at the same time. Here we noticed that for a good detection scheme of 90%, we will need from 3 dB to 6 dB of more gain of signal-to-noise ratio. This is by comparing with the orthogonal wave, and depending on the chosen channel. With the use of Algorithms 1 and 2, we can choose the interference channel that leads to a least deterioration of the radar wave and produce a better output by improving the execution of the detection. At the same time, minimizing additional gain in SNR is needed. In this instance, the two algorithms (1 and 2) will choose BS3 and because of this condition, the NSP wave needs the lowest gain of signal-to-noise ratio to reach a perfection detection, probably over 90 percent in comparison to the other base stations.

Another scenario is with dim $\mathbb{N}\left(H_{i}\right)$ = 6, as shown in Figure 3. We presented a performance of five different NSP signals scheme, but in this instance the MIMO radar has a bigger antenna set if we compare it to the earlier scenario. In the present scenario, to obtain the best signal detection probability of 90%, we will have to reach 2.3 to 3.3 dB of more gain on SNR in comparison to orthogonal wave. Similar to the earlier scenario, by applying Algorithms 1 and 2, we will be able to choose an interference channel scheme that leads to a minimum deterioration of radar wave. This shows a high performance of target detection scheme with the lowest additional gain in SNR of interest. In addition, Algorithms 1 and 2 will choose Base Station No. 4. This is because the NSP waveform needs a very low gain of signal-to-noise ratio performance detection sequence of 0.9%, if compared with other base stations. These two cases show the significance of Algorithms 1 and 2 on projection probability of the radar by proving its performance in the selection of the channel. That leads to a least interference scheme and improves the coexistence of the two communications systems (Radar–Mobile LTE). This reduces significantly the gain in signal-to-noise ratio needed for null space projection of radar waves.

**Figure 4:**
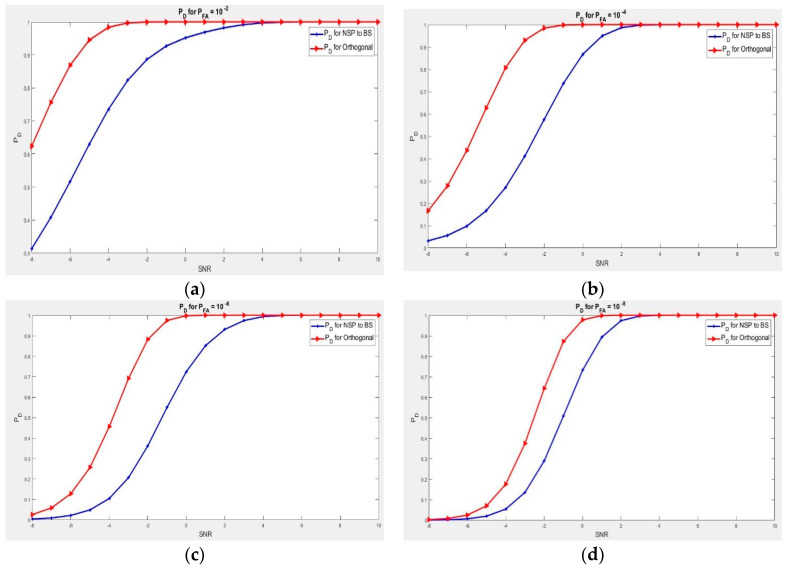
(**a**) Detection probability for SNR. Dimension $\mathbb{N}\left(H_{i}\right)$ = 2, $P_{f}$ = $10^{-2}$, and 3.6 dB is needed to have the same result with orthogonal waveform; (**b**) Detection probability for SNR. Dimension $\mathbb{N}\left(H_{i}\right)$ = 2, $P_{f}\;$= $10^{-4}$, and 4.1 dB is needed to have the same result with orthogonal waveform; (**c**) Detection probability for SNR. Dimension $\mathbb{N}\left(H_{i}\right)$ = 2, $P_{f}\;$= $10^{-6}$, and 2.9 dB is needed to have the same result with orthogonal waveform.; (**d**) Detection probability for SNR, Dimension $\mathbb{N}\left(H_{i}\right)$ = 2, $P_{f}\;$= $10^{-8}$, and 1.8 dB is needed to have the same result with orthogonal waveform.

In Figure 4, we show the alterations of probability of detection $P_{D}$ in the form of SNR for different probability of false alarm $P_{f}$. Every figure shows the probability of detection $P_{D}$ for a fixed point; $P_{D\;}against\;P_{f}$ has been evaluated for $10^{-2}$,$\;10^{-4}$,$\;10^{-6}$,$\;10^{-8}$, respectively, with the dimension 2 × 4 of interference channel, which implies antennas radar M = 4, and $N^{BS}$ = 2 for base station antennas, with the dimension $\mathbb{N}\left(H_{i}\right)$ = 2 of null-space.

While comparing the sensing operation, the observation of the two waveforms brings clarity. In order to get a better detection probability in a fixed point of $P_{f}$, we will need more gain level for signal-to-noise ratio for the null space project if we compare it with the orthogonal waves. By considering $P_{D\;}$= 0.9 as best detection probability or 90%, we will need 1.8 to 4.1 dB of extra gain on the null space project waveform in order to perform similar results with the orthogonal wave signal.

In the first scenario, as observed, the two signal waveforms increased simultaneously, where the signal-to-noise ratio increases. Despite this, comparing the two waves for a chosen point of signal-to-noise ratio, we can observe that orthogonal signal waves showed a very good performance compared to the null space projection wave. This means that the waves transmitted are no longer orthogonal, which also means that we can no longer get the benefit of orthogonal waves when we are using massive multi-input multi-output radar, as we have highlighted in Section 3.1. The good news is that we have canceled all interferences at the base station level. In addition, in Scenario 1 as shown in Figure 2, for us to execute the needed signal detection for a settled $P_{f}$, we will need more signal-to-noise ratio for null space projection as compared with Figure 4. This is due to many radar antennas that we used, while the antennas of the base station remain at the same point in Figure 4, which increases the aspect of the NSP channel. This results in a very good signal operation for null space projection wave. As we are interested in reducing the bad impact of the null space projection on radar in order to optimize its operation, it is better to make use of more radar antennas transmitter array.

### 3.2. Analysis of Scenario 2

Here, for every run of the Monte Carlo simulation, we generate interference channel with Ҡ Rayleigh signal. Each sequence has a dimension of Ҡ$N^{BS}\times M$. We compute null spaces and generate matching matrix projection according to Algorithm 4, and project the radar signal detection by applying Algorithm 3. We transmit the NSP signal detection, evaluate the parameters θ and α from the receiver, and estimate orthogonal and NSP waves signal detection.

**Figure 5:**
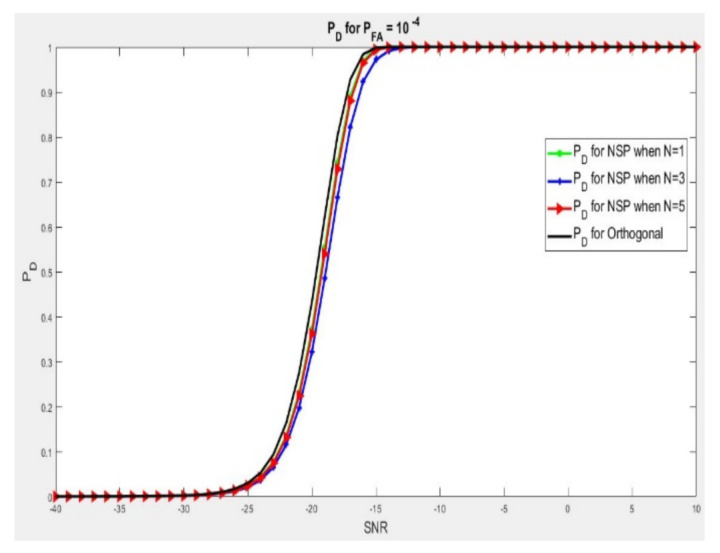
$P_{D}$ at SNR where $P_{f}\;$= $10^{-4}$ and massive radar lessens disturbance in the network at all the base stations where *M* = 100, Ҡ = 4, and N = {1,3,5}.

In Figure 5, we have a scenario where the radar wave signal has a considerable antenna array, if we compare it with Ҡ mobile base stations antenna array. In such a case, we have so much degree of freedom (DoF) for a good target detection at the radar and at the same time canceling interference to all base stations present in the network. Here we examined M = 100, Ҡ = 4, and N = {1,3,5}. We analyzed $P_{D}$ against $P_{f}\;$= $10^{-4}$ for a mixed disturbances channel H, considering the dimensions Ҡ$N^{BS}\times M$, where we equalize signal detection performance of primary wave and the null space projection wave inside channel interferences. It was observed that for us to obtain the probability of detection that we need for a fixed probability of false alarm, we will need more signal-to-noise ratio compared to the orthogonal waves. If we need a detection performance of $P_{D}$ = 0.90, we need 0.6, 1 and 2.5 dB of extra signal-to-noise ratio for the null space projection wave, where N is 1, 5 and 3 accordingly to obtain exactly the same result.

## 4. Discussion

Cognitive radio-based spectrum sharing is a new opportunity to face spectrum shortage in a world where everything tends to convert into a co-existence sharing. In this study, we analyzed spatial technique on spectrum access for massive MIMO radar and massive mobile communication systems, by considering numerous numbers of base stations. Our objective here is the cancellation of all interference schemes on the space. The selection of the best cancellation channel is made possible based on the maximum estimation and projection algorithm scheme, for a complete cancellation of disturbance to the cooperative radio frequency of existence. 

1st Projection Algorithms Scenario, where M <Ҡ$N^{BS}$ but M > $N^{BS}$: In this scenario, spectrum is spitted on setting up a projection matrix, then choosing an interference channel which is carried by the support of Algorithms 1 and 2. Primarily, on each and every impulse recurrence time (IRT), the radar receives its SSCI for the whole Ҡ intrusion channels. The prediction matrix is created by the data transmitted through Algorithm 2 and the process of null spaces. In the first Algorithm, we showed the prediction arrays which are represented by Ҡ, obtained based on Algorithm 2, to get the forecast matrix which leads to minimum deterioration of radar waves and it is typical to improve the system. The present action goes alongside with the null space of chosen base stations through radar waves for matching chosen projection array and waveform transfer.
**Algorithm 1**. Projection for Algorithm 1 on 1st Scenario.Iterate For *i* = 1: Ҡ;  Obtain SSCI of $H_{i}$ from the replay of $i^{th}$ Antenna   Forward $H_{i}$ to Algorithm 2 for projection matrix generation   $P_{i}$.end for Learn $i_{min}$=$\;\arg\underset{1\leq i\leq Ҡ}{\max}\begin{array}{c} {\left|\left|P_{i}x\left(t\right)-x\left(t\right)\right|\right|}_{2} \end{array}$
Define $P_{i_{min}}=\overset{ˇ}{P}$ to be the desired projection.Implement the null space projector: $\overset{ˇ}{x}\left(t\right)=\overset{ˇ}{P}x\left(t\right)$
End 

2nd Projection Algorithm Scenario, where M > $ҠN^{BS}$: Here, the spectrum sharing is performed throughout Algorithm 3 and 4. Initially, we considered that at each impulse recurrence time (IRT), SSCI of the total Ҡ interference channels has been received from the radar. And the data are forwarded to Algorithm 4 for the computation of null space which will result in H and create the matrix projection we called P. Algorithm 3 performs radar’s wave projection into the null space.
**Algorithm 2**. Projection for Algorithm 2 on 1st Scenario.If Algorithm 1 received interferences $H_{i}$, next Execute $H_{i}$ ⇒ $H_{i}=U_{i}\;\Sigma_{i}\;V_{i}^{H}$
Construct $\tilde{\Sigma_{i}}=diag\left(\tilde{\sigma_{i1}},\tilde{\sigma_{i2}},\ldots ,\tilde{\sigma_{ip}}\right)$
Construct ${\tilde{\Sigma_{i}}}^{\prime }=diag\left({\tilde{\sigma_{i1}}}^{\prime },{\tilde{\sigma_{i2}}}^{\prime },\ldots ,{\tilde{\sigma_{iM}}}^{\prime }\right)$
Setup Projection Matrix $P_{i}=V_{i}\;{\tilde{\Sigma_{i}}}^{\prime }\;V_{i}^{H}$
Send $P_{i}$ to Algorithm 1. End 
**Algorithm 3**. Projection Algorithm 3 on 2nd Scenario.Iterate By observation from Ҡ base stations obtain SSCI of *H*.  Forward Interference *H* to Algorithm 4 and create matrix P.  After Receiving matrix projection *P* through Algorithm 4.  Execute zero interference projection,$\;\overset{ˇ}{x}\left(t\right)=Px\left(t\right)$
End 
**Algorithm 4**. Projection Algorithm 4 on 2nd Scenario.If Algorithm 3 has sent information to *H*, next  Execute *H* ⇒ $H\;=U\;\Sigma \;V^{H}$
  Build $\tilde{\Sigma }=diag\left(\tilde{\sigma_{1}},\tilde{\sigma_{2}},\ldots ,\tilde{\sigma_{p}}\right)$
  Build ${\tilde{\Sigma_{i}}}^{\prime }=diag\left({\tilde{\sigma_{1}}}^{\prime },{\tilde{\sigma_{2}}}^{\prime },\ldots ,{\tilde{\sigma_{M}}}^{\prime }\right)$
  Determine Matrix Projection $P=V\;{\tilde{\Sigma }}^{\prime }\;V^{H}$
  And matrix P is forwarded to Algorithm 3.End 

## 5. Conclusions

In the future, spectrum sharing of radar radio frequency will be definitely shared with advanced evolution mobile communication systems (without mentioning the huge demand of Internet of Everything). This will curtail the escalation of bandwidth demand and reduce bad consequences of spectrum blockage for business and commercial communications platforms. In this paper, we studied a comparative spectrum-sharing scheme for radars and LTE mobile communications systems. An approach based on spatial technique was proposed to reduce signal interference of the radar at the mobile cellular communication environment. Our attention was more on reducing interferences on the radar side, where our target was to cancel and eliminate all forms of interferences, especially from the radar scheme, in a way that there is no more source of disturbance to the mobile base station of interest. We have expanded the concept by projecting signal detection of one radar system to null space interference channel of LTE mobile communication with numerous BSs. The parameter of a chosen target point was estimated and we trained the detection performances of the spectrum sharing for massive multi-input multi-output radars. We generated a statistical sensing detection estimation for targeted sensing point. We also applied the generalized likelihood ratio test (GLR) for determining whether the target point is present or not, while applying orthogonal waves and null space projection waves. The suggested spectrum-sharing algorithm can be applied in different scenarios, where massive multi-input multi-output of the radar is sharing spectrum environment with mobile LTE communications, by canceling and minimaxing all deterioration schemes in its operation.

## Figures and Tables

**Table 1:** Summary table of Notations.

Notations	Values
$x\left(t\right)$	Radar Wave Transmitted
$a_{T}\left(\theta \right)$	Steering Vector Transmitted of Target angle *θ*
$a_{R}\left(\theta \right)$	Steering Vector Received of Target angle *θ*
$A\left(\theta \right)$	Transmit-Receive Steering Matrix
$y\left(t\right)$	Received Radar Wave
$R_{x}$	Matrix of Correlation
$s_{i}^{\mathrm{UE}}$(t)	$\mathrm{Transmitted}\;\mathrm{signal}\;\mathrm{of}\;j^{th}$ $\;\mathrm{UE}\;\mathrm{in}\;i^{th}BS$
$L_{i}^{\mathrm{UE}}$	$\mathrm{Total}\;\mathrm{Number}\;\mathrm{of}\;\mathrm{UEs}\;\mathrm{in}\;i^{th}BS$
Ҡ	Total Number of BSs
M	Radar Transmit Antenna
$N^{\mathrm{BS}}$	BS Transmit/Receive Antenna
$H_{i}$	$\mathrm{Interference}\;\mathrm{Channel}\;\mathrm{between}\;\mathrm{Radar}\;\mathrm{and}i^{th}BS$
$H_{i,j}$	$\mathrm{Channel}\;\mathrm{between}\;j^{th}UE$ $\;\mathrm{and}\;i^{th}BS$
$r_{i}\left(t\right)$	$\mathrm{Received}\;\mathrm{Signal}\;\mathrm{at}\;i^{th}BS$
$P_{i}$	$\mathrm{Projection}\;\mathrm{Matrix}\;\mathrm{for}\;\mathrm{the}\;i^{th}\mathrm{Channel}\;$

**Table 2:** Massive MIMO Radar Parameters for Test Environment.

Parameters	Notations	Values
Radar & LTE Communication RF Band	-	3550–3650 MHz
Radar Antenna Tx/Rx	*M*	10/4
LTE Communication System Antennas	$N^{\mathrm{BS}}$	5
Carrier Frequency	f	3.55 GHZ
Wavelength	λ	8.5 cm
Antenna Inter Spacing	3λ/4	6.42 cm
Radial Velocity	$v_{r}$	1000 m/s
Speed of Light	c	3 × $10^{8}$ m/s
Target point	$r_{o}$	400 Km
Angle of Target	θ	$\hat{\theta }$
Doppler Frequency	$\omega_{D}$	2$\omega_{c}v_{r}$/c
Two-way breeding holdup,	$\tau_{r}$	2$r_{o}$/c
Path loss	α	$\hat{\alpha }$

## Data Availability

Data can be delivered upon request by reaching out through the corresponding emails.
